# Individualized Physiotherapy of Upper Body Functional Movement Disorder – Two Illustrative Cases

**DOI:** 10.5334/tohm.895

**Published:** 2024-06-28

**Authors:** Christof Degen-Plöger, Annemarie Reincke, Christina Bolte, Carl Alexander Gless, Kerstin Luedtke, Alexander Münchau, Kirsten E. Zeuner, Anne Weissbach

**Affiliations:** 1 Institute for Health Sciences, Department of Physiotherapy, University of Lübeck, Lübeck, Germany; 2 Institute for Sports Science, Faculty of Philosophy, Christian-Albrechts-Universität of Kiel, Kiel, Germany; 3 Institute of Systems Motor Science, University of Lübeck, Lübeck, Germany; 4 Department of Neurology, Christian Albrechts-University of Kiel, Kiel, Germany; 5 Center of rare diseases, University Clinic Schleswig-Holstein, Lübeck, Germany; 6 Center of Brain, Behavior and Metabolism, University of Lübeck, Lübeck, Germany

**Keywords:** Physiotherapy, functional movement disorder, attention training

## Abstract

**Background:**

Information on specialist physiotherapeutic treatment for functional movement disorders is scarce. Previous studies focussed on functional gait disorders and availability of descriptions of the practical application especially for other body regions is very limited.

**Cases:**

We present two illustrative cases, demonstrating the key elements of physiotherapy for the treatment of functional movement disorders beyond gait difficulties. The individual applicability of the specific core elements of physiotherapy, adapted to the individual needs of each patient, are described. We also explain, how different sensory stimuli can be used to shift attention away from symptoms and thus reduce them. Moreover, we discuss how patients’ agency can be encouraged and how this results in therapy key moments, contributing to a sustained improvement of symptoms.

**Conclusion:**

Thus, our case series are intended to guide clinicians and therapists alike, to promote disease-specific physiotherapy for this common and treatable neuropsychiatric disorder.

## Introduction

Functional neurological disorders are common and multiple neurological professions are engaged in their management [1]. One of the largest subgroups of functional neurological disorders are motor/movement disorders. Despite their great heterogeneity, they are all characterized by inconsistency in severity and phenomenology under attentional refocusing. As a second diagnostic pillar incongruency to other non-functional movement disorders and neuroanatomical rules has been proposed. However, there is an intensive discussion whether this term should be avoided, as it might just be another way of making a diagnose of exclusion and requires omniscience in clinical neurology [2]. Unfortunately, many patients are undiagnosed or have long diagnostic delays and thus receive no or delayed therapy, which is mostly unspecific [1]. Based on the biopsychosocial model, functional movement disorders are multifactorial in origin, which makes it essential for the specific physiotherapy to identify individual factors. Predisposing vulnerabilities, e.g. in the biological context of an “organic” disease, in the sense of an emotional disorder on a psychological level, or in the social context of a specific life event or difficulty. Precipitation mechanisms, e.g. in the biological context of a physical injury/pain or e.g. in the psychological context of a life event perceived as negative and unexpected. Perpetuating factors are described in the biological context as plasticity in the motor and sensory pathways of the CNS leading to habitual abnormal movements and deconditioning, or in the psychological context as abnormal perceptions of illness [3]. These factors influence the development and progression of the disorder and their processing helps to design a comprehensive and individualized therapy together with the patient. Therefore, specific physiotherapy follows a cognitive behavioral approach and differs from conventional neurological therapy concepts [3]. Of note, specifically targeted physiotherapy is considered a first-line treatment [4,3,5] and greatly differs from conventional physiotherapy concepts for neurological diseases [1]. However, only a few studies [3,6,7,8] provided information on specific symptoms, e.g., functional tremor [7], but detailed descriptions and practical or real-world applications for the physiotherapeutic treatment of functional movement/motor disorders that go beyond functional gait disorders or affect the upper body are mostly missing.

For this reason, we here describe two illustrative cases demonstrating the applicability of previous findings and their adaptation to functional disorders other than gait disorders.

## Case Settings

Based on two illustrative cases, we demonstrate how specific physiotherapy can be practically applied in an outpatient setting for patients with functional movement disorders of the face and upper body. Patients received between 10 and 20 treatment sessions, each lasting approximately 60 minutes. Core elements of the specific physiotherapy were adapted from consensus recommendations [3] and included 1) psychoeducation about the clinical picture to promote its acceptance and understanding, 2) redirecting attention to foster movement (re)learning by promoting automated movements and 3) establishing awareness of self-efficacy of symptom reduction. A detailed summary of the clinical and therapeutic characteristics of both patients is given in Table 1.

**Table 1:** Clinical and therapeutic characteristics of patients.


CLINICAL AND THERAPEUTIC CHARACTERISTICS OF PATIENTS	CASE 1	CASE 2

**Main symptoms**	Weakness of eyelid opening Perioral jerks	Postural and intention tremor left arm

**Secondary symptoms**	Fatigue	Fatigue Pain in neck, shoulders

**Work**	Unable to work 6 months before her first therapy session at the age of 20	Receiving disability pension since the age of 55

**Age at onset**	19	52

**Age at diagnosis**	20	55

**Age at intervention**	21	58

**Previous treatment**	Physiotherapy with craniosacral therapy Botulinum neurotoxin i.m. injections	Manual therapy with massages of neck muscles

**Individualized movement treatment with specific individualized modalities/stimuli**	Personal preference and interest in learning to juggle, e.g., gamification Refocusing of attention away from eyelid closure towards autonomic driven function with juggle Personal key moment by recognizing the function in the video feedback Motivated and informed to practice a home exercise training	Reflexion of everyday life Recognition of the symptom triggers and their dependence Personal key moment when recognizing the reversibility of the symptoms Focus on directed attention with haptic and auditive stimulus e.g., texture of the knife handle when cutting, listening the bubbles when drinking, rhythmic foot tapping Building self-confidence through symptom control, being back to society

**Goals pre-treatment**	Voluntarily opening eyes: 10 min. sports 1–2 hrs. riding horse 1–2 hrs. computer work	Cooking, cutting vegetables without symptoms Reviving social activities

**Goals post-treatment**	2–3 times per week fitness Functioning at work	Attending concerts, exhibitions with more self-confidence and trust


## Case 1 «Eyes on the juggle»

### Background

A 21-year-old woman presented with weakness of eyelid opening and perioral jerks (Video 1a) since the age of 19 years. The diagnosis of a functional movement disorder was made at the age of 20 years. Before commencing specific physiotherapy, she was on sick leave from her job as a hearing aid acoustician for six months. She also stopped doing sports (fitness and yoga) as this had caused worsening of symptoms. Only during horse riding, an improvement of eyelid opening was observed by friends, which, however, was not perceived by the patient herself.

**Video 1a:** Pre-intervention. **1b:** Start schematic juggling (in front of a mirror). **1c:** Key moment during physiotherapy (in front of a mirror). **1d:** Progression in juggling. **1e:** Post-intervention.

### Intervention

At the start of therapy, the patient defined her primary treatment goal as voluntary opening and controlling of her eye movements especially during sports activities and computer work. She was instructed about the clinical picture and possible improvement using the example of horse riding, which improved symptoms by redirecting attention. In a next step, we introduced juggling to the patient, which she always wanted to learn. Through its motivating, playful character it very efficiently redirected her attention away from the affected body regions (eyes, mouth). Initially, a schematic structure of juggling was instructed, e.g., throwing and catching one ball per hand and throwing from one side to the other (Video 1b). Juggling was performed in front of a mirror and videotaped, so the patient received direct visual feedback. Recognizing that she could reduce her symptoms by directing attention towards catching the ball was perceived as a key moment, i.e., as if she “flipped a switch in her head” and she felt empowered (Video 1c). In addition to her physiotherapy sessions twice a week, she practiced two times per week by herself. In the third week of treatment, she was symptom-free. From then on, she could manage her private and professional life without symptoms. Next, we gradually made juggling more difficult, e.g., using three balls, or juggling while standing on a wobble board, until the end of therapy (Video 1d & 1e).

In addition, we encouraged the patient to identify perpetuating factors, e.g., her working conditions. Thus, additional relaxation strategies such as grounding [5] and pacing [3] were practised. After further improvement of her performance and increasing confidence, the patient decided to resume her professional work and initiated regular fitness training and yoga in addition to her hobby of horse riding.

More than a year after the end of treatment, the patient is still symptom-free. She identified her stressful working condition as a disease perpetuating factor. Thus, she changed her job and recently started working as a horse caretaker.

## Case 2 «Back to society with touch and hearing»

### Background

A 58-year-old woman was suffering for six years from postural and intention tremor of the left hand. The tremor was accompanied by fatigue and pain in the neck and shoulders. She quit her job, had difficulties performing household activities and could no longer pursue hobbies. The tremor increased with conscious use of the arm or when trying to supress it (Video 2a). She realized that routine tasks not requiring much attention, e.g., cleaning herself in the bathroom, sometimes considerably ameliorated the tremor. Due to the variability of the symptoms and their rapid change she felt insecure during activities. Moreover, she had difficulty explaining her symptoms to her family and friends, which resulted in loss of social contact.

**Video 2a:** Pre-intervention. **2b.** Key moment during physiotherapy. **2c.** Post-intervention.

### Intervention

In the first sessions, the understanding of the clinical picture, its therapy, and the importance of conscious control of attention were discussed. The above-mentioned examples from everyday life were used to illustrate the attention dependency of symptoms and their variability. The patient quickly recognised additional triggering factors such as crowds and the associated inner expectation that “nothing will work anymore”. Together we defined therapy goals as reducing the tremor during cooking and eating and being able to meet family and friends again. During the first treatment sessions, the patient realized that her tremor decreased when she focused attention on the texture of objects she was using, e.g., the perception of the texture of a cup when drinking or the handle of a knife when chopping. Sounds the patient could focus on when moving her arm were also helpful, e.g., her tremor decreased while concentrating on the sounds of the bubbles when drinking sparkling water. Focusing on rhythmic motor performance, for example when tapping her foot on the ground, was also helpful to gain control of her arm movements (Video 2b & 2c). The patient’s own perception of the change was supported by video feedback to visualize the reversibility of symptoms. This was perceived as a key moment. Her self-confidence grew and she started to incorporate techniques she had learned into her daily life, e.g., while attending concerts and family celebrations. Moreover, she used the knowledge she gained about the disease to explain her symptoms to family members and friends, which had previously hindered her from meeting them.

Six months after the treatment, the patient was able to willingly control her tremor, even though the symptoms persisted in her daily life. It seemed that the patient only used her treatment strategies, when necessary, i.e., when the tremor appeared. Meaning that these strategies are used more as compensation rather than a consistent learning method to adapt motor control. At the same time, from the authors’ perspective, this suggests an incomplete understanding of the illness and its therapy, as there was also a lack of engagement with potential disease-perpetuating factors in her daily life.

## Discussion

Our case series demonstrate that disease-specific physiotherapy in an outpatient setting is feasible and effective in patients with various functional movement disorders of the face and upper body. Such treatment differs from conventional physiotherapy applied for non-functional neurological disorders, with its core element being not to focus on dysfunction/ affected limbs. Thus, manual therapy or strength training, focussing on movement execution and control of the affected body region, are inefficient and can instead maintain and even worsen symptoms [3]. In contrast, physiotherapy for functional movement disorders educates patients to control and redirect attention away from the symptomatic body part, thus reducing excessive pathological extrinsic control and fostering more automatic, intrinsic movements [3,5,4]. For this purpose, at the beginning of our training, we analysed which stimulus quality, e.g., acoustic, tactile, or visual, helped the individual patient best to redirect her attention easily and effectively away from the symptomatic body region.

Moreover, our treatment comprised psychoeducation. We enabled the patients to realize that there are certain mechanisms in their daily life that alleviated their symptoms (e.g., hobbies) and included them in our treatment. Therefore, interests and personal preferences should be integrated into treatment sessions when designing individual therapeutic strategies. With the help of visual feedback (e.g., mirror, video), patients experienced their agency, which increased their self-confidence and encouraged them to increase their training level, i.e., trying out new motor tasks. Patients reported key moments resembling turning points in coping with the disease. Importantly, patients described that our training not only reduced motor symptoms but also had, albeit less so, positive effects on fatigue, mood, concentration, and pain.

It is important to keep in mind that in our experience patients with functional movement disorders tend to over-perform once they notice symptom reduction. Newly gained control of action can lead to increased expectations, pressure, and stress, which in turn can result in relapses. In such situations focussing on psychoeducation is very helpful. Thus, the physiotherapist can enable the patient to identify disease mechanisms that might have contributed to the current symptom exacerbation and ensure that the patient fully understood the disease and its perpetuating factors. Moreover, including pacing, graded exercise, and relaxation strategies can help to better cope with psychological stressors [3,5,6,9].

Another aspect for relapse of symptoms could be that stimuli and training techniques became too trivial to effectively capture attention and require adaptation to keep the attention span high enough. Thus, a gradual increase of performance difficulty is recommended.

Our case series resemble two different responses to specialized physiotherapeutic treatment. The first patient totally recovered from symptoms whereas the second one used the treatment to temporarily suppress her symptoms. Thus, the latter patient continued to have symptoms especially when confronted with disease-triggering factors during her daily life. It emphasizes that a central understanding and acceptance of the illness and its therapy are key mechanisms to achieve consistent symptom relief. Therefore, it is crucial to facilitate interdisciplinary exchanges with other disciplines involved in the treatment of the same patient. It is highly recommended to ensure a uniform treatment according to the same guidelines. A broad range of information and training for therapists and other healthcare professionals is needed to raise awareness of functional neurological disorders and to establish nationwide therapeutic services.

## Financial Disclosures for the previous 12 month

**Christof Degen-Plöger**

**Table:** 


Stock Ownership in medically related fields	Christof Degen-Plöger has no stock ownership in medically related fields

Intellectual Property Rights	Christof Degen-Plöger has no intellectual property rights

Consultancies	Christof Degen-Plöger has no consultancies

Expert Testimony	Christof Degen-Plöger has no function as an expert testimony

Advisory Boards	Christof Degen-Plöger is not a member of any advisory board

Employment	University Medical Center Schleswig-Holstein, Campus Lübeck

Partnerships	Christof Degen-Plöger does not enter partnerships

Inventions	Christof Degen-Plöger has no inventions

Contracts	Christof Degen-Plöger has no contracts

Honoraria	Christof Degen-Plöger does not receive any honoraria

Royalties	Christof Degen-Plöger receives no royalties

Patents	Christof Degen-Plöger owns no patents

Grants	Christof Degen-Plöger does not receive any grants

Other	Christof Degen-Plöger declare that there are no additional disclosures to report


**Annemarie Reincke**

**Table:** 


Stock Ownership in medically related fields	Annemarie Reincke has no stock ownership in medically related fields

Intellectual Property Rights	Annemarie Reincke has no intellectual property rights

Consultancies	Annemarie Reincke has no consultancies

Expert Testimony	Annemarie Reincke has no function as an expert testimony

Advisory Boards	Annemarie Reincke is not a member of any advisory board

Employment	University Medical Center Schleswig-Holstein, Campus Kiel

Partnerships	Annemarie Reincke does not enter partnerships

Inventions	Annemarie Reincke has no inventions

Contracts	Annemarie Reincke has no contracts

Honoraria	Annemarie Reincke does not receive any honoraria

Royalties	Annemarie Reincke receives no royalties

Patents	Annemarie Reincke owns no patents

Grants	Annemarie Reincke does not receive any grants

Other	Annemarie Reincke declares that there are no additional disclosures to report


**Christina Bolte**

**Table:** 


Stock Ownership in medically related fields	Christina Bolte has no stock ownership in medically related fields

Intellectual Property Rights	Christina Bolte has no intellectual property rights

Consultancies	Christina Bolte has no consultancies

Expert Testimony	Christina Bolte has no function as an expert testimony

Advisory Boards	Christina Bolte is not a member of any advisory board

Employment	University Medical Center Schleswig-Holstein, Campus Lübeck

Partnerships	Christina Bolte does not enter partnerships

Inventions	Christina Bolte has no inventions

Contracts	Christina Bolte has no contracts

Honoraria	Christina Bolte does not receive any honoraria

Royalties	Christina Bolte receives no royalties

Patents	Christina Bolte owns no patents

Grants	Christina Bolte does not receive any grants

Other	Christina Bolte declares that there are no additional disclosures to report


**Carl Alexander Gless**

**Table:** 


Stock Ownership in medically related fields	Carl Alexander Gless has no stock ownership in medically related fields

Intellectual Property Rights	Carl Alexander Gless has no intellectual property rights

Consultancies	Carl Alexander Gless has no consultancies

Expert Testimony	Carl Alexander Gless has no function as an expert testimony

Advisory Boards	Carl Alexnader Gless is not a member of any advisory board

Employment	University Medical Center Schleswig-Holstein, Campus Kiel

Partnerships	Carl Alexander Gless does not enter partnerships

Inventions	Carl Alexander Gless has no inventions

Contracts	Carl Alexander Gless has no contracts

Honoraria	Carls Alexander Gless does not receive any honoraria

Royalties	Carl Alexander Gless receives no royalties

Patents	Carl Alexander Gless owns no patents

Grants	Carl Alexander Gless does not receive any grants

Other	Carl Alexander Gless has been supported by the Faculty of Medicine, University of Kiel, Germany.


**Kerstin Lüdtke**

**Table:** 


Stock Ownership in medically related fields	Kerstin Lüdtke has no stock ownership in medically related fields

Intellectual Property Rights	Kerstin Lüdtke has no intellectual property rights

Consultancies	Kerstin Lüdtke has no consultancies

Expert Testimony	Kerstin Lüdtke has no function as an expert testimony

Advisory Boards	Kerstin Lüdtke is not a member of any advisory board

Employment	University of Lübeck

Partnerships	Kerstin Lüdtke does not enter partnerships

Inventions	Kerstin Lüdtke has no inventions

Contracts	Kerstin Lüdtke has no contracts

Honoraria	Kerstin Lüdtke reports honoraria from Novartis Pharma for Migraine Workshops (€1500)

Royalties	Kerstin Lüdtke receives no royalties

Patents	Kerstin Lüdtke owns no patents

Grants	Kerstin Lüdtke declare grants from Versorgungssicherungsfond for migraine app development (€370.000 for 2023–20205)

Other	Kerstin Lüdtke declare that there are no additional disclosures to report


**Alexander Münchau**

**Table:** 


Stock Ownership in medically related fields	Alexander Münchau has no stock ownership in medically related fields

Consultancies	Alexander Münchau has consultancies for PTC Therapeutics

Advisory Boards	Alexander Münchau is on advisory board for German Tourette syndrome Association and Alliance of patients with chronic rare diseases

Partnerships	Alexander Münchau does not enter partnerships

Inventions	Alexander Münchau has no inventions

Honoraria	Alexander Münchau reports honoraria from Desitin, Teva and Takeda

Grants	Alexander Münchau declare support from Foundations: Possehl-Stiftung (Lübeck, Germany), Margot und Jürgen Wessel Stiftung (Lübeck, Germany), Tourette Syndrome Association (Germany), Interessenverband Tourette Syndrom (Germany), CHDI, Damp-Stiftung (Kiel, Germany); Academic research support: Deutsche Forschungsgemeinschaft (DFG): projects 1692/3-1, 4-1, SFB 936, and FOR 2698 (project numbers 396914663, 396577296, 396474989); European Reference Network – Rare Neurological Diseases (ERN – RND; Project ID No 739510)

Intellectual Property Rights	Alexander Münchau has no intellectual property rights

Expert Testimony	Alexander Münchau has no function as an expert testimony

Employment	University of Lübeck; University Medical Center Schleswig-Holstein, Campus Lübeck

Contracts	Alexander Münchau has no contracts

Royalties	Alexander Münchau reports royalties for the book *Neurogenetics* (Oxford University Press)

Patents	Alexander Münchau owns no patents

Other	Alexander Münchau declare commercial research support from: Pharm Allergan, Ipsen, Merz Pharmaceuticals, Actelion


**Kirsten E. Zeuner**

**Table:** 


Stock Ownership in medically related fields	Kirsten E. Zeuner has no stock ownership in medically related fields

Intellectual Property Rights	Kirsten E. Zeuner has no intellectual property rights

Consultancies	Kirsten E. Zeuner has served as a consultant and received fees from Ipsen, Alexion, Zambon and the German Federal Institute for Drugs and Medical Devices (BfArM).

Expert Testimony	Kirsten E. Zeuner has no function as an expert testimony

Advisory Boards	Kirsten E. Zeuner has served on advisory boards for Ipsen, AbbVie, Bial, Alexion, Merz

Employment	Kirsten E. Zeuner is an employee of the University Medical Center Schleswig Holstein, Campus Kiel

Partnerships	Kirsten E. Zeuner does not enter partnerships

Inventions	Kirsten E. Zeuner has no inventions

Contracts	Kirsten E. Zeuner has no contracts

Honoraria	Kirsten E. Zeuner reports speaker’s honoraria from Bayer Vital GmbH, BIAL, Alexion, AbbVie and Merz outside the submitted work

Royalties	Kirsten E. Zeuner receives no royalties

Patents	Kirsten E. Zeuner owns no patents

Grants	Kirsten E. Zeuner has received research support from the Christa and Hans-Peter Thomsen Foundation, the German Research Foundation (DFG 5919/4-1) and from Strathmann GmbH & Co. KG.

Other	Kirsten E. Zeuner declares that there are no additional disclosures to report


**Anne Weissbach**

**Table:** 


Stock Ownership in medically related fields	Anne Weissbach has no stock ownership in medically related fields

Intellectual Property Rights	Anne Weissbach has no intellectual property rights

Consultancies	Anne Weissbach has no consultancies

Expert Testimony	Anne Weissbach has no function as an expert testimony

Advisory Boards	Anne Weissbach is not a member of any advisory board

Employment	University Medical Center Schleswig-Holstein, Campus Lübeck

Partnerships	Anne Weissbach does not enter partnerships

Inventions	Anne Weissbach has no inventions

Contracts	Anne Weissbach has no contracts

Honoraria	Anne Weissbach does not receive any honoraria

Royalties	Anne Weissbach receives no royalties

Patents	Anne Weissbach owns no patents

Grants	Anne Weissbach declares grants from: German Research Foundation (DFG, WE5919/2-1, WE 5919/4-1, and FOR2698/2), the Dystonia Medical Research Foundation, and an Edmond J. Safra Career Development Award from the Michael J. Fox foundation (MJFF-022062)

Other	Anne Weissbach declares that there are no additional disclosures to report


## References

[1] Popkirov S. Funktionelle neurologische Störungen. Erkennen, verstehen, behandeln. Springer eBook Collection (1st ed. 2020. ed., pp. 1 Online-Ressource (IX, 213 S. 232 Abb.)). Berlin, Heidelberg: Springer; 2020. DOI: 10.1007/978-3-662-61272-9

[2] Stone J. Incongruence in FND: time for retirement. Pract Neurol. 2024; 24(2): 163–165. DOI: 10.1136/pn-2023-003897 38212112

[3] Nielsen G, Stone J, Matthews A, Brown M, Sparkes C, Farmer R, et al. Physiotherapy for functional motor disorders: a consensus recommendation. J Neurol Neurosurg Psychiatry. 2015; 86(10): 1113–1119. DOI: 10.1136/jnnp-2014-309255 25433033 PMC4602268

[4] Aybek S, Perez DL. Diagnosis and management of functional neurological disorder. BMJ. 2022; 376: o64. DOI: 10.1136/bmj.o64 35074803

[5] Maggio J, Kyle K, Stephen CD, Perez DL. Lessons Learned in Outpatient Physical Therapy for Motor Functional Neurological Disorder. J Neurol Phys Ther. 2023; 47(1): 52–59. DOI: 10.1097/NPT.0000000000000415 35980727

[6] Nielsen G. Physical treatment of functional neurologic disorders. Handb Clin Neurol. 2016; 139: 555–569. DOI: 10.1016/B978-0-12-801772-2.00045-X 27719871

[7] Espay AJ, Edwards MJ, Oggioni GD, Phielipp N, Cox B, Gonzalez-Usigli H, et al. Tremor retrainment as therapeutic strategy in psychogenic (functional) tremor. Parkinsonism Relat Disord. 2014; 20(6): 647–650. DOI: 10.1016/j.parkreldis.2014.02.029 24679736 PMC4028378

[8] LaFaver K, Espay AJ. Diagnosis and Treatment of Functional (Psychogenic) Parkinsonism. Semin Neurol. 2017; 37(2): 228–232. DOI: 10.1055/s-0037-1601487 28511263

[9] Gilmour GS, Nielsen G, Teodoro T, Yogarajah M, Coebergh JA, Dilley MD, et al. Management of functional neurological disorder. J Neurol. 2020; 267(7): 2164–2172. DOI: 10.1007/s00415-020-09772-w 32193596 PMC7320922

